# Rare diseases' genetic newborn screening as the gateway to future genomic medicine: the Screen4Care EU-IMI project

**DOI:** 10.1186/s13023-023-02916-x

**Published:** 2023-10-04

**Authors:** Alessandra Ferlini, Edith Sky Gross, Nicolas Garnier, on behalf of the Screen4Care consortium, Joanne Berghout, Aldona Zygmunt, Deependra Singh, Kui A. Huang, Waltraud Kantz, Carl Rudolf Blankart, Sandra Gillner, Jiawei Zhao, Richard Roettger, Christina Saier, Jan Kirschner, Joern Schenk, Leon Atkins, Nuala Ryan, Kaja Zarakowska, Jana Zschüntzsch, Michela Zuccolo, Matthias Müllenborn, Yuen-Sum Man, Liz Goodman, Marie Trad Lysogene, Anne Sophie Chalandon, Stefaan Sansen, Maria Martinez-Fresno, Shirlene Badger, Rudolf Walther van Olden, Robert Rothmann, Patrick Lehner, Christof Tschohl, Ludovic Baillon, Gulcin Gumus, Rumen Stefanov, Georgi Iskrov, Ralitsa Raycheva, Kostadin Kostadinov, Georgi Stefanov, Elena Mitova, Moshe Einhorn, Yaron Einhorn, Josef Schepers, Miriam Hübner, Frauke Alves, Rowan Iskandar, Rudolf Mayer, Alessandra Renieri, Aneta Piperkova, Ivo Gut, Sergi Beltran, Mads Emil Matthiesen, Marion Poetz, Mats Hansson, Regina Trollmann, Emanuele Agolini, Silvia Ottombrino, Antonio Novelli, Enrico Bertini, Rita Selvatici, Marianna Farnè, Fernanda Fortunato

**Affiliations:** 1 Medical Genetics Unit, Department of Medical Sciences, University of Ferrara, 44121 Ferrara, Italy; 2 EURORDIS – Rare Diseases Europe, Paris, France; 3 Pfizer Inc., Collegeville, 19426 PA USA

**Keywords:** Rare diseases, Genetic newborn screening, Next generation sequencing, Digital health, European Union

## Abstract

Following the reverse genetics strategy developed in the 1980s to pioneer the identification of disease genes, genome(s) sequencing has opened the era of genomics medicine. The human genome project has led to an innumerable series of applications of omics sciences on global health, from which rare diseases (RDs) have greatly benefited. This has propelled the scientific community towards major breakthroughs in disease genes discovery, in technical innovations in bioinformatics, and in the development of patients’ data registries and omics repositories where sequencing data are stored. Rare diseases were the first diseases where nucleic acid-based therapies have been applied. Gene therapy, molecular therapy using RNA constructs, and medicines modulating transcription or translation mechanisms have been developed for RD patients and started a new era of medical science breakthroughs. These achievements together with optimization of highly scalable next generation sequencing strategies now allow movement towards genetic newborn screening. Its applications in human health will be challenging, while expected to positively impact the RD diagnostic journey. Genetic newborn screening brings many complexities to be solved, technical, strategic, ethical, and legal, which the RD community is committed to address. Genetic newborn screening initiatives are therefore blossoming worldwide, and the EU-IMI framework has funded the project Screen4Care. This large Consortium will apply a dual genetic and digital strategy to design a comprehensive genetic newborn screening framework to be possibly translated into the future health care.

## The path toward genomic medicine

The human genome project, via an innumerable series of applications of omics sciences on global health, has led important implications in rare diseases (RDs) [1]. Among these, the Online Mendelian Inheritance in Man (OMIM, https://omim.org), is now a free repository which lists all known mendelian genes in which pathogenic variations cause RDs [2]. Besides, several RD registries and datasets are available worldwide providing huge data sets of patient’s phenotypes and genotypes [3, 4].

This RD intensive research, accompanied by the development of new therapies, prompts for updated newborn screening strategies, to gain early diagnosis by population screening in all neonates in a defined geography. Since the first Guthrie test for phenylketonuria (PKU) in 1961, biochemical NBS for many treatable RDs have become available, although differently adopted by health systems in various countries across the world [5]. Biochemical tests were further implemented by tandem mass spectrometry (MS/MS) and are currently performed on screening blood spot cards. Metabolic screening had an immense impact on patients by early detection at a relatively low cost and revolutionized the natural history of many RD types. Nevertheless, significant limitations remain. First, metabolic NBS can be adopted only for diseases which have disease-specific analytes; second, it explores phenotypes, implying that genetic diagnosis must be subsequently very often carried out for disease confirmation; and third, conflicting results are not infrequent [6]. Consequently, only a limited number of RDs are currently identifiable by metabolic NBS and can benefit from this extraordinary screening tool. Several initiatives are ongoing to explore the feasibility of genetic NBS (gNBS) on a large-scale, using different next-generation sequencing (NGS) approaches [7–12]. The international Consortium (ICoNS, https://www.iconseq.org) was thoughtfully established to enhance collaboration and data sharing among these initiatives.

Broad implementation of gNBS holds tremendous promise, but also raises important ethical questions. As in routine genetic diagnosis, gNBS needs to meet quality standards, such as accuracy (identifying the variant correctly), sensitivity (probability to detect the variations), specificity (ability to not identify the variations in non-carriers) and clinical validity (ability to predict a specific phenotype) [13].

Different techniques could be adopted, including targeted sequencing (or TS, also defined as ‘on demand’ gene panels), whole exome sequencing (WES), or whole genome sequencing (WGS). While TS is validated and widely used in diagnostics, WGS might start to gradually replace TS, as soon as WGS can fulfil the standards mentioned above, in addition to possibly becoming more cost effective than TS [14–16].

gNBS must be able to identify all mutation types, including copy number variations (CNVs), to confer full accuracy to the testing; robust data pipelines for variants' calling prioritization and validation are needed, and gNBS for not deferrable RDs (eligible for interventions or therapy in a certain, not flexible, temporal window) needs appropriate reporting turnaround time. All these issues encourage the use of large and high-throughput platforms able to process thousands of samples to secure timely access to care.

Irrespective of the techniques we may prefer to adopt for gNBS, selecting genes/diseases to be screened and how and to whom reports shall be communicated is a crucial task. Following the EURORDIS document listing the 11 key principles to be considered for NBS (https://www.eurordis.org/publications/key-principles-for-newborn-screening), we need to "decrypt” the definitions of treatable and actionable conditions, before finding rigorous rules for their inclusion in gNBS. The next critical step is reaching some level of consensus on how to build robust genes/diseases selection criteria. An arbitrary disease selection based on technique popularity or availability, or on subjective preference from any stakeholder, not evidenced based, will be unsuccessful. In addition, the RD advocacy community will need to provide evidence that proposed gNBS programs meet the needs of patients. It will be crucial finding a consensus about minimal requirements needed for gNBS adoption in health systems, depending on geographies, cultures, preferences, and means. Related to this last point, the large genetic datasets generated in diverse ethnicities will provide unique information about population-specific clinical utility of gNBS for certain diseases, fact that will possibly address the more appropriate methods to be used. Finally, if gNBS will be adopted by health systems, an unprecedented amount of data will be generated, raising outstanding issues related to data privacy, ethics, and ownership, and how to make optimal and appropriate use of them both in research and healthcare contexts [17].

## Screen4Care: an EU-IMI funded project for RDs early diagnosis

The European Commission (EC), through its Innovative Medicines Initiative (IMI) has funded the research project Screen4Care (www.screen4care.eu). This five-year long project, launched in October 2021, is based on a “private public patient partnership” or “PPPP” including 36 partners working together in a coordinated way to define, develop, and pilot the “optimal” comprehensive framework for gNBS to be proposed as part of a future healthcare. The EC has dedicated an impressive amount of funding into RD research, with an established and successful track record of delivering results. It is therefore not at all surprising that EC has deemed it important to invest in such a project oriented towards genetic newborn screening and digital health. Indeed, Screen4Care (S4C) will use a multi-pronged strategy to shorten the time to diagnosis for RD patients. Firstly, it is undertaking an ecosystem systematic scoping of all significant initiatives on RDs' diagnosis and genetic NBS in Europe. This will establish a S4C RD federated meta data platform. A second pillar of the project is running a “real life” gNBS in about 25,000 infants in Europe, the largest population ever genetically screened before [18], using custom, “ad hoc” designed,  gene panels. Disease prioritization will be conceptualized around treatable diseases, after having defined what “treatable” means. This will be achieved by delineating a robust scoring method which will be used to identify “treatable” diseases and then applied on a broader yet specific actionable disease spectrum, this last enriched by insights from a large public survey conducted by EURORDIS. The S4C gNBS work package also includes design of gNBS pipeline(s) adapted to the participating countries, from preference studies to technical setting, ethical considerations, also using telegenetics and telemedicine. Among the 25,000 screened infants, those manifesting early symptoms of a RD (any) within a 1–2 year follow up, though resulted negative at the genetic panel screening, will receive whole genome sequencing WGS. In addition, S4C will develop several digitals tools based on Artificial Intelligence-trained algorithms to identify phenotypes in patients at early disease onset and to help them in their diagnosis journey. S4C pillars will be complemented by cost/benefit evaluation, enabling S4C to put forth technically, ethically, and economically sustainable gNBS framework.

S4C is rooted in sharing and dialoguing, especially with the NBS community to harmonize metabolic and genetic NBS approaches, to contribute to a larger consensus on the most effective NBS-gNBS strategies and other early diagnosis models, adaptable to context-specific environments and health systems. We underline that the involvement in the S4C of the RD community with the active role of EURORDIS as a full partner, represents an added value since it will allow to define views and perceptions of society about gNBS, as already recommended worldwide [19].

We believe, as it happened in the Human Genome project, that defining gNBS appropriate path(s) for RDs will benefit both health system and genomic medicine future avenues, representing a pioneering global health approach.

## Data Availability

S4C website contains additional data and materials www.screen4care.eu

## Abbreviations

RD: Rare disease
gNBS: Genetic newborn screening
S4C: Screen4Care
WGS: Whole genome sequencing
WES: Whole exome sequencing
TS: Targeted sequencing
CNV: Copy number variation
